# Parasites of the Human Body

**Published:** 1856-07

**Authors:** A. Henry


### PARASITES OF THE ANIMAL BODY.*

ORDINARY observation, as well as the researches of scientific men, have rendered familiar to us the fact, that certain animals assume different forms in different stages of their existence. Everyone, for instance, knows that the tadpole and the frog are in reality one and the same animal, and that the terms caterpillar, chrysalis, and butterfly, merely denote different stages of existence of the same being. But, besides these well known instances, naturalists have become aware of the circumstance, that certain of the lower forms of animal life, which were, when first noticed, believed to be distinct species and even genera, are in reality but transitional stages of animals, which may even be remarkably different from them both in external shape and in habits. Numerous examples of this have been found, for example, in the crustacea, or that tribe to which the crab and lobster belong.

Such researches as these in relation to disease may at first sight seem merely interesting—more fanciful than useful. But, in regard to one class especially of animal beings, the discovery of the fact that some of them are merely transitional forms of others, is likely to be of the greatest importance as indicating the means of preventing the recurrence of

---

* VON SIEBOLD, Karl Theodor, Ueber die Band und Blasenwürmer, nebst einer Einleitung über die Entstehung der Eingeweidewürmer. (On Tape and Vesicular Worms: with an introduction on the Production of Intestinal Worms.) Leipzig: 1154.

THOMSON, Allen, M.D., F.R.S., Notice of Recent Researches on the Origin of Entozoa, more especially of Tape-worms. In *Glasgow Medical Journal*, July 1855.

BARKER, T. Herbert, M.D., On Cystic Entozoa in the Human Kidney. London: 1856. Hamilton.

JORDAN, R. C. R., M.B., Lecture on the Entozoa. In *Association Medical Journal*, August 31, 1855.

a very common class of diseases. The demonstration of this fact will now be our task.

There is a well-known class of beings, of simple form, whose distinctive peculiarity is that they exist within or on the bodies of other animals. Hence they have received the name of *entozoa* (*ἐντός*, within; *ζῶον*, an animal) or parasites. In popular language, they are called intestinal worms. It may be remarked in passing, that some parasites, from their being attached to external parts, as the eyes of fishes, have received the name of *epizoa* (*ἐπί*, on; *ζῶον*, an animal); but this distinction is more artificial than natural, and, for all practical purposes in this place, it is sufficient to confine our remarks to the entozoa, or those animals which exist within the body.

The entozoa have been arranged, according to their forms, into four classes: 1. The cystic or bladder-shaped worms; 2. The cestoid or tapeworms; 3. The trematode worms; 4. The hematoid or thread-worms.

1. The *cystic* or bladder-shaped worms, also commonly called hydatids, are characterised by possessing a round (or nearly round) more or less transparent body, distended with fluid matter, and having a projecting part or head, surrounded by one or more rows of curved spines or hooklets —thus bearing some resemblance to a poppy-head. The most common animals of this group, with their ordinary places of habitation, are:

The *echinococcus hominis*, or liver hydatid, principally found in the liver of man, sometimes also in the kidneys, spleen, heart, brain, blood, or bones.

The *cysticercus cellulosæ*, usually found in the tissue between the muscles of man, also sometimes in the eyes, heart, liver, and other organs. It is frequent in the flesh of pigs, where it produces the disease called measles. The *cysticercus pisiformis*, or pea-shaped cysticercus, a small variety, inhabits the body of the rabbit. The *cysticercus fasciolaris* is found in the liver of the rat and mouse. The *cysticercus tenuicollis* inhabits domestic animals.

The *cœnurus cerebralis* is ordinarily found in the brains of sheep, where it produces the disease termed "staggers."

2. The *cestoid* or tapeworms have the body composed of a number of segments jointed together, giving the appearance from which their popular name is derived. The head is furnished with a series of small mouths or suckers, and with a ring of hooklets, by which they are enabled to maintain their hold of the animal on which they live. The principal forms of cestoid worms are the following.

The *tænia solium*, or ordinary tapeworm, inhabits the human intestines, and is of frequent occurrence. The *tænia crassicollis* commonly dwells in the intestine of the cat. The *tænia serrata* infests the intestines of dogs.

The *bothriocephalus latus*, or broad tapeworm, occurs in the human body in eastern Germany, Russia, and Switzerland.

Certain other cestoid worms, known by the names of *rhynchobothrius* and *ligula*, are met with in fishes, and in sea-fowl and other animals which feed on them.

3. The *trematode* worms are represented by the *distoma hepaticum*, or common fluke, which inhabits the liver of sheep, producing the disease called "rot."

4. The *nematoid*, or thread-worms, so called from their small size, are represented by the following genera.

The common *ascaris* inhabits the lower part of the intestinal canal. Some ascarides, as the *ascaris incisa*, found in the peritoneal cavity of the mole, are enclosed in membranous cysts or bags.

The *filaria medinensis*, or Guinea-worm, is common in tropical countries, especially on the coast of Africa, and infests the tissue under the skin of the body, generally the leg.

It will be seen that the entozoa are found in two classes of situations: viz., in the surface of the alimentary canal, or in the interior of organs, as the liver, lungs, brain, heart, kidneys, blood, and beneath the skin, in the muscular tissue, etc. Of the two forms with which we principally have to deal here—the cystic and the cestoid worms—the former inhabit the interior of organs, while the latter attach themselves to the alimentary canal. This is an important fact to be remembered; for out of it has in a great degree grown the inquiry, whether these cystic and cestoid worms are really distinct from each other, or whether the cystic is merely an early stage of development of the cestoid. If the latter supposition be true, then the cystic worm can only become cestoid by being transferred to a suitable habitation; and one of the most obvious ways is, by being eaten in the flesh of the animal infected with it, and thus transferred to the alimentary canal of another animal. That this is what actually takes place, is what modern researches have proved.

The principal investigators of this subject have been Professor von Siebold of Munich; Eschricht of Copenhagen; Van Beneden; Küchenmeister of Zittau; Dujardin; Blanchard, as well as those other observers whose names are placed at the beginning of this article. The researches of Küchenmeister are of special interest and importance.

The main point which has been made out, and which has a most important bearing on public health, is this: that the cystic worms are but embryonic or early conditions of the cestoid; and that man and other animals, by eating meat in which cystic entozoa exist, are liable to become infested with cestoid or tape-worms. The condensed narrative of a few of the experiments which have been performed will show what this means.

1. Ten young dogs were fed by Von Siebold with the *cysticercus pisiformis* (or pea-shaped hydatid) from the rabbit. They were killed and opened at different successive periods afterwards, when the gradual process of the conversion of the cysticerci into tæniæ or tape-worms was observed in the intestines.

2. Six young dogs were fed with the *cysticercus tenuicollis*, which is common in domestic cattle. Dr. Von Siebold gave only the heads of the animals. The result was exactly the same as in the former case, tape-worms being developed, which reached their full development in forty-eight days.

3. Four young dogs had given to them at different times, in their food, the *cysticercus cellulosæ* from the flesh of the hog. On their being opened at different intervals afterwards, there were found in the intestines, in various stages of development, tape-worms which exactly resembled the *tænia serrata*. Von Siebold was struck with the close resemblance of the *tænia serrata* of dogs to the *tænia solium* of man, and believes that they are identical, or at most only varieties of the same species, the difference being dependent on their habitations.

4. A similar experiment was performed by Von Siebold with the heads of the *cœnurus cerebralis*, the entozoon which infests the brains of sheep and cattle, producing staggers. The dogs experimented on were carried to a part of the country where a number of the sheep were affected with sturdy. In the intestines of five out of seven dogs fed with the cœnurus, great numbers of tape-worms were found at successive periods, in different degrees of advancement.

5. Von Siebold gave to twelve young dogs and a fox quantities of echinococcus animalcules in milk. On their being examined at various periods, up to thirty-six days, there was found, in all stages of development, a small tape-worm, totally different from any described in the previous experiments, or indeed from any one hitherto accurately distinguished.

6. Dr. Küchenmeister, having previously caused the production of the serrated tape-worm in the dog by feeding it with the *cœnurus cerebralis* from a sheep, gave to young lambs some of the joints of the tape-worm, with the effect of producing by the fifteenth day the usual symptoms of sturdy. The same results were obtained by Van Beneden at Louvain, Eschricht at Copenhagen, Leuckart at Giessen, and Haubner at Dresden.

6. Leuckart had in his possession a family of white mice, which he had employed for various experiments, and in none of which had the *cysticercus fasciolaris,* which infests the liver of these animals, been found. He gave to six out of twelve, with their food and drink, the ova of the *tænia crassicollis* or tape-worm of the cat. Four months afterwards he found, on opening these mice, that four of them were affected with the *cysticercus fasciolaris* of the liver ; while in none of the mice which had not received the ova of tape-worm was there any trace of hydatids.

7. A most interesting experiment was performed by Dr. Küchenmeister, on a condemned criminal. He gave the man, at seven successive times, between a hundred and thirty and twelve hours previous to his execution, a number of *cysticerci* from the hog, and some from the rabbit, mingled with various articles of food. On examination after death, a number of young tape-worms, in different stages of development, were found in the intestine of the man.

8. Some experiments of Küchenmeister, Van Beneden, and Haubner, show that the *cysticercus cellulosæ* may be produced in great quantity in hogs by feeding these animals with joints of the common tape-worm of man ; but that this does not occur in the dog or sheep. The experiments of Küchenmeister further tend to show that each form of hydatid—*cysticercus, cœnurus,* or *echinococcus*—produces its own form of tape-worm, and no other ; and *vice versâ.*

9. M. Van Beneden brought up two newly-born puppies under the same conditions, except that to one of them a certain number of *cysticerci* were administered in his food, while these worms were carefully kept from the second. The *cysticerci* were administered on March 12 and 23, and on April 21. These dogs were killed and opened on April 25, when the animal which had eaten no *cysticerci* was quite free from the *tænia serrata;* while the intestines of the other dog contained three bundles of worms, which were considered to be the *tænia serrata.* M. Van Beneden has repeated experiments of this kind a number of times with the same results.

10. The interesting case narrated by Dr. Barker of Bedford, in the paper the title of which is placed at the beginning of this article, was that of a patient, in whom quantities of *echinococci* used to escape from his body with the renal secretion. On inquiring into the nature of this man's food, Dr. Barker obtained the following interesting information :—

"For some years past he has rarely eaten either beef or mutton, having a natural aversion to these meats, and for one year, six years ago, he was a vegetarian. As an ordinary rule, however, he has lived on pork, and thinks that, on an average, he has taken 'pig's fry', consisting principally of the liver, at least twice weekly. He has on more than one occasion eaten 'measly' pork, and pig's chitterlings (the intestines of the animal) has been a frequent dish. He is also very fond of sheep's head, and especially of the brains, but does not know whether the brains he has thus taken were those of 'sturdy' sheep. He has likewise been accustomed to take in the morning herbal bitters, such as decoctions of horehound, wormwood, and agrimony. He is fond of coarse brown sugar. He does not remember ever having eaten meats badly cooked, and has not suffered from other forms of entozoa, except ascarides, which troubled him greatly in early life. His wife (since their marriage) has lived on the same diet, but has not shown symptoms of the same disease."

11. In the instance of a woman, in many respects similar to the case just referred to, Mr. Evans, of St. Neot's, gave Dr. Barker the following information with regard to the food of the patient :—

"In regard to diet, I have ascertained from my patient, that, about seventeen years since, she, as well as the whole of the family, were much in the habit of eating pig's brains in large quantity, as well as occasionally pig's fry; but that, since her first symptoms of disorder, now ten years ago, she has lived principally on mutton. The statement she made was, that her father, being a waggoner, was in the habit of bringing home large pigs' heads. Her mother usually put the brains into a pudding with seasoning, to constitute a meal for the family, and they individually ate heartily of it. No other instance of hydatids were known in the family."

These experiments, which are but a few among many which have been performed, demonstrate that an entozoon resident in one animal is transmissible to the body of another by being administered with the food. Thus a cystic worm may produce a cestoid or tape-worm; and, on the other hand, a cestoid worm may give origin to a cystic. Further, it appears that any form is not produced indiscriminately from another, but that each cystic has its own representative cestoid.

Cases of the kind narrated by Dr. Barker suggest the question—how is it that a person who has taken cystic worms in his food, sometimes has cystic worms—not cestoid—developed in his body? The answer to this appears to lie in the fact, that the mode of development of the entozoa is mainly dependent on the part of the body which they reach. Those entozoa which are merely attached to the intestinal canal become cestoid or tape-worms, while those which are confined in the structure of organs, as the liver, kidney, brain, etc., retain the cystic form.

An argument in favour of the connexion of the occurrence of tape-worm in the human body with the eating of uncooked meat, is afforded by the well known fact regarding the Abyssinians, who are currently reported to consume large quantities of raw meat, and in whom tape-worm is a common affection. It is to be hoped that investigations will be made into the amount and nature of entozoic disease to which animals in Abyssinia are subject.

Dr. Von Schleisner, in his *Medical Topography of Iceland*, informs us that the inhabitants of that country have long been suffering from an hydatid disease—the hydatids affecting the liver, peritoneum, and tissue under the skin. About one-sixth of the whole population is said to be affected by it. Professor Von Siebold believes it probable that this disease depends on the introduction of the ova of a tape-worm into the body; and that this arises from the immense quantity of dogs kept in Iceland for herding sheep and cattle. This question, however, is open to further investigation.

The demonstration of the communicability of entozoa from one animal to another is of great importance in regard to the preservation of health. The development of worms in the human body is always recognised as at least a troublesome disease, and often as one attended with considerable impairment of the physical powers of the patient. To meet this evil, we have been ransacking the stores of curative medicine; and purgatives, turpentine, pomegranate, kousso, fern oil, and other drugs without end, have had each their trial. But in this instance, as in others, "prevention is better than cure"; and it is to be trusted that the knowledge of such facts as we have related, supported as they are by the evidence of trustworthy observers, will lead to the exercise of a greater amount of care in regard to the use of animal food. To the poor, who are accustomed to use those parts of animals most liable to be infested with entozoa, and who are less careful than they ought to be in subjecting their meat to a sufficient culinary process, this information is second to none in a sanitary point of view.

*A. Henry.*

---

### THE PHYSICAL EDUCATION OF WOMEN.*

